# Transport regimes of a split gate superconducting quantum point contact in the two-dimensional LaAlO_3_/SrTiO_3_ superfluid

**DOI:** 10.1038/s41467-018-04657-z

**Published:** 2018-06-11

**Authors:** Holger Thierschmann, Emre Mulazimoglu, Nicola Manca, Srijit Goswami, Teun M. Klapwijk, Andrea D. Caviglia

**Affiliations:** 1 Kavli Institute of Nanoscience, Faculty of Applied Sciences, Delft University of Technology, Lorentzweg 1, 2628 CJ Delft, The Netherlands; 2 QuTech, Delft University of Technology, Lorentzweg 1, 2628 CJ Delft, The Netherlands; 3 Physics Department, Moscow State University of Education, Moscow, 119991 Russia

## Abstract

One of the hallmark experiments of quantum transport is the observation of the quantized resistance in a point contact in GaAs/AlGaAs heterostructures. Being formed with split gate technology, these structures represent in an ideal manner equilibrium reservoirs which are connected only through a few electron mode channel. It has been a long standing goal to achieve similar experimental conditions also in superconductors. Here we demonstrate the formation of a superconducting quantum point contact (SQPC) with split gate technology in a two-dimensional superconductor, utilizing the unique gate tunability of the superfluid at the LaAlO_3_/SrTiO_3_ interface. When the constriction is tuned through the action of metallic split gates we identify three regimes of transport: First, SQPC for which the supercurrent is carried only by a few quantum transport channels. Second, superconducting island strongly coupled to the equilibrium reservoirs. Third, charge island with a discrete spectrum weakly coupled to the reservoirs.

## Abstract (web summary)

Quantum transport in superconductors remains difficult to study due to the typically small Fermi wavelength. Here, Thierschmann et al. demonstrate a superconducting quantum point contact with split gate technology at the superconducting LaAlO_3_/SrTiO_3_ interface and, due to its two-dimensionality, identify three regimes of quantum transport.

## Introduction

Ever since the seminal experiments by van Wees et al.^1,2^ on a quantum point contact formed with split gates in a semiconductor heterostructure, it has been a great experimental challenge to achieve similar experimental conditions also in superconductors^3^. This is generally desirable because the split gates enable quantum transport experiments within one and the same electronic system, without the need to combine different material systems. Such structures ideally represent equilibrium reservoirs which are connected only through a quantum constriction with a set of conducting channels, each of which has a certain transmission probability, as envisioned in the Landauer-Büttiker picture of quantum transport. With superconductors such conditions were accomplished only in atomic scale mechanically tunable break junctions of conventional superconducting metals, but here the Fermi wavelength is so short that it leads to a mixing of quantum transport with atomic orbital physics^4^.

Split gates further allow for convenient in situ control of sample properties such as Fermi wavelength and the shape of the confinement potentials for charge carriers. Various attempts have therefore been made to combine the desired gate-tunability of the low electron density semiconductor with the use of conventional superconductors. However, these hybrid devices have introduced, compared to the GaAs/AlGaAs normal quantum transport case, the very important and yet very difficult to control influence of the interface between the two dissimilar materials^5^. This makes the results dependent on the complexities of the proximity effect and thus complicates their interpretation.

**Fig. 1:**
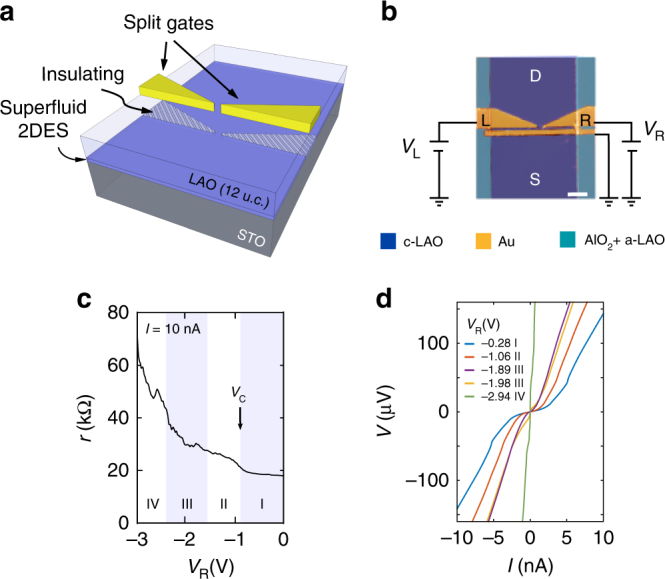
Two-dimensional superconductor with split gates. **a** Generalized sketch of the device. At the interface between the STO substrate and the 12 unit cell (u.c.) layer of crystalline LAO (c-LAO) the superconducting 2DES (blue) is formed, which can be tuned insulating (shaded blue) locally under the split gates (yellow), thus forming a superconducting constriction. **b** False color atomic force microscope image showing the device layout. The potential of the split gates (yellow) L and R is controlled with the voltages *V*_L_ and *V*_R_, respectively. *V*_L_ is kept at −1 V. The conductive 2DES is formed in regions with c-LAO (blue). In areas which are protected with an AlO_2_ hard mask LAO growth is amorphous (a-LAO, turquoise). The thin gate spanning the channel is not used in the experiments. It is therefore kept at ground potential. Scale bar = 1 μm. **c** High bias (*I* = 10 nA) differential resistance *r* as a function of *V*_R_. I, II, III, and IV indicate the different regimes of transport (see text). *V*_c_ denotes the formation of the constriction. **d** *I*–*V* curves for different gate voltages *V*_R_ with *V*_L_ = −1 V. I–IV refer to the different regimes of transport indicated in Fig. 2. For regime III two curves are shown with a high and a low zero bias resistance, respectively

In principle, a new path has become available when it was discovered that in the two dimensional electronic system (2DES) at the LaAlO_3_–SrTiO_3_ (LAO–STO) interface superconductivity becomes suppressed when the electron density *n* is reduced below a critical value *n*_c_, for example, by means of a gate voltage^6,7^. The Fermi-wavelength *λ*_F_ in this system can be as large as 30–50 nm^8^ and ballistic transport in the normal state has been demonstrated^8,9^. The superconducting coherence length is about *ξ* = 100 nm^10,11^. This corresponds to spatial dimensions which are commonly achieved with present day lithography techniques. The creation of a superconducting quantum point contact (SQPC) with split gates in LAO/STO should therefore be within reach [cf. Fig. 1a]. In contrast to previous results on hybrids and mechanical break junctions, which used conventional bulk superconductors, it is to be expected that the two-dimensionality of the LAO/STO superfluid will play a significant role for the outcome of such an experiment. This approach can also offer insight into the nature of superconducting pairing at oxide interfaces. Unconventional pairing was recently suggested^12,13^ in light of a number of experimental observations, including strong spin orbit coupling^14^, co-existence of ferromagnetism and superconductivity^15–17^, indications for electron pairing without macroscopic phase coherence^8,18–20^ and a non-trivial relation between the critical temperature *T*_c_ and charge carrier density *n*^6,20^.

Here we present experiments that demonstrate the formation of a SQPC with split gates in the LAO/STO superfluid. We find that the quantum constriction undergoes different regimes of transport when tuned through the action of the split gates. While for more open configurations the supercurrent is carried by a single transport mode, a charge island is formed when the constriction becomes pinched off. The island energy spectrum is dominated by Coulomb repulsion and it exhibits a superconducting ground state when it is coupled more strongly to the reservoirs while for weaker coupling discrete electronic energy states appear.

## Results

### Split gate control of a quantum constriction

Our samples are fabricated following the procedure described by Goswami et al.^7^ (see Methods section and Supplementary Table 1). The measurements discussed in the main text were obtained from a single device, further measurements from a second sample are provided in the Supplementary Note 7. Figure 1b presents a false color atomic force microscope image of the device layout. The metallic split gates (yellow) L and R cover the full width of the 5 μm wide 2DES (blue), except for a 150 nm region at its center. Transport experiments are performed in a current bias configuration (unless stated otherwise) at temperature *T*_base_ < 40 mK. The resistively measured transition to the superconducting state is observed at *T*_c_ ≈ 100 mK (see Methods section and Supplementary Fig. 2). Because of gate history effects, we carry out the experiment by putting electrode L on a fixed gate voltage (*V*_L_ = −1 V) to ensure depletion and tune the constriction by only varying the voltage *V*_R_ applied to gate R (see Supplementary Note 6).

We expect the following scenario: When *V*_R_ is changed toward negative values the charge carrier density *n* gets reduced locally underneath the gate and gets closer to the critical density *n*_c_ at which superconductivity becomes suppressed. At a certain gate voltage *V*_R_ = *V*_c_ the condition *n* = *n*_c_ is reached and a supercurrent can flow only through the constriction between the tips of the gates, thus forming a weak link between the superconducting reservoirs. Outside this weak link, under the gates, the system acts as an insulator^6^. The number of transport modes available in the weak link is determined by its effective width. The constriction width is reduced when *V*_R_ is further decreased and therefore the number of transmission channels decreases which is expected to lead to a step-wise reduction of the critical current *I*_c_^3^. For $V_{\mathrm{R}} \ll V_{\mathrm{c}}$ transport will be dominated by a low transmissivity and the current is pinched off.

**Fig. 2:**
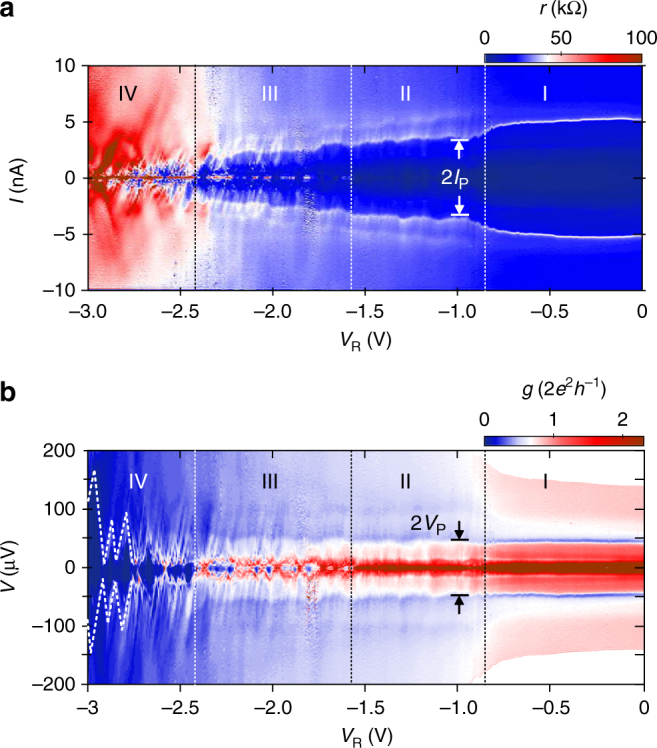
Transport regimes in the constriction. **a** Differential resistance *r* versus current *I* for gate voltage *V*_R_ = 0 to −3 V. Gate L is kept at *V*_L_ = −1 V. The four regimes of transport I to IV are indicated. *I*_P_, reminiscent of the critical current *I*_c_ of the weak link, is indicated. **b** Same data as in **a** but with voltage drop *V* on the vertical axis and differential conductance *g* represented by the color scale. *V*_P_ denotes the voltage drop at *I*_P_. Dotted lines in regime IV indicate conductance diamonds

In order to study this scenario, we record a series of *V*–*I* curves and vary *V*_R_ from 0 to −3 V. Panoramic overviews of the results are given in Fig. 2a, b in color plots. Figure 2a presents the differential resistance *r* = d*V*/d*I* and Fig. 2b shows the differential conductance *g* = d*I*/d*V* with the current *I* and voltage drop *V* on the vertical axis, respectively. It can be seen that the constriction undergoes four different regimes of transport (labelled I–IV in the figures) as *V*_R_ is varied from 0 to −3 V. Representative *I*–*V* curves from each regime are shown in Fig. 1d. Regime I (ranging from *V*_R_ = 0 to −0.9 V) corresponds to the open current path configuration with *V*_R_ > *V*_c_. A sharp peak in *r* is visible at *I* = ±5 nA, labelled *I*_P_ in Fig. 2a, which is reminiscent of a critical current *I*_c_. Correspondingly, a dip occurs in *g* at *V*_p_ = ±44 μV (Fig. 2b). At *V*_R_ = *V*_c_ ≈ −0.9 V the critical density *n*_c_ is reached. Here *I*_P_ drops significantly because the current path becomes confined. At high currents in Fig. 2a, as shown for *I* = 10 nA in Fig. 1c, this point of confinement is apparent in a step increase in *r*, similar to the well-known behavior in semiconductor heterostructures^21^. It marks the transition to regime II (*V*_R_ = −0.9 to −1.6 V). In this regime *I*_P_ decreases when *V*_R_ is reduced indicating the gate tunable weak link. In regime III (*V*_R_ = −1.6 to −2.4 V) regions of high resistance at zero bias appear and disappear periodically. As we will show below, this can be attributed to the emergence of a conductive island which dominates transport through the constriction. In regime IV (*V*_R_ = −2.4 to −3 V) the device always exhibits a high resistance at zero bias. Figure 2b reveals that this regime is controlled by conductance diamonds (CDs) (indicated with dashed lines).

### Superconducting quantum point contact

**Fig. 3:**
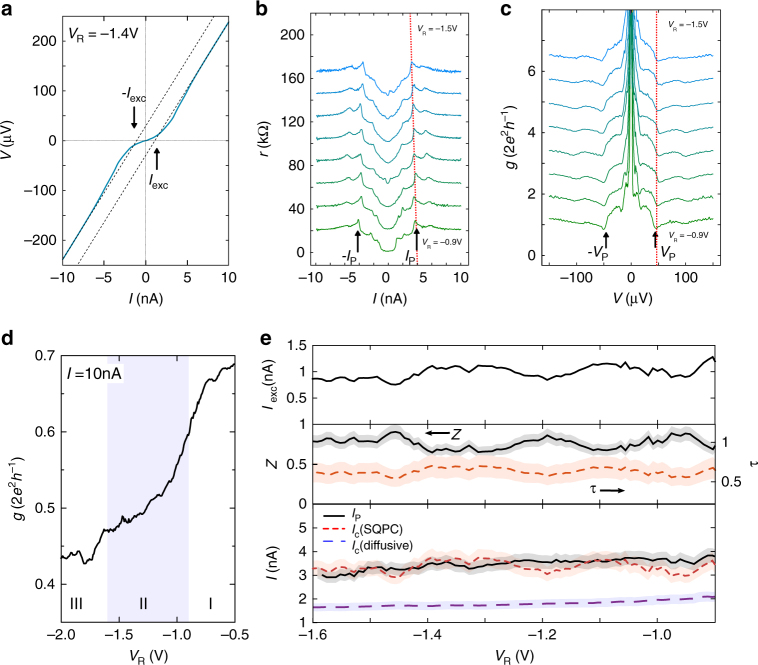
Superconducting quantum point contact in regime II. **a** Representative *V*–*I* curve in regime II (*V*_R_ = −1.4 V). The rounded supercurrent is clearly visible. The excess current is denoted *I*_exc_. **b** *r* versus *I* for different *V*_R_. *I*_P_, inferred from the sharp peak in *r*, is indicated. The change of *I*_P_ with *V*_R_ is highlighted by a dashed line as guide-to-the-eye. The curves are offset by 10 kΩ. **c** *g* versus *V* for different *V*_R_. *V*_P_ ≈ 44 μV is indicated with arrows and a dashed line as guide-to-the-eye. The curves are offset by 0.2 (2*e*^2^*h*^−1^). **d** *g* for *I* = 10 nA. **e** top panel: *I*_exc_ as a function of *V*_R_. Middle panel: Barrier strength *Z* (solid, black line) and normal state transmission *τ* (dashed, red line) as obtained from BTK. Bottom panel: *I*_P_ (solid, black line) and critical current *I*_c_ as expected for a SQPC (short, red dashes). For comparison, *I*_c_ expected for a diffusive junction with conductance *g* is shown (blue dashes). Shaded regions indicate the standard deviation

Let us start with the weak link regime II. Here we observe a rounded supercurrent and an excess current *I*_exc_ ≈ 1 nA [Fig. 3a]. *I*_P_, reminiscent of the critical current *I*_c_, changes from 3.7 to 3.0 nA [Fig. 3b] when *V*_R_ is varied. The voltage *V*_P_ = 44 μV [cf. Fig. 3c] can be related to the superconducting gap *V*_P_/2 ≈ Δ ≈ 22 μeV, which is compatible with the value inferred from the resistively measured *T*_c_, Δ_*T*c_ = 1.76*k*_B_*T*_c_ = 15 μeV. The high bias conductance *g*_n_ [Fig. 3d] is of the order of half the quantum of conductance, changing with *V*_R_ from 0.6 to 0.47 (2e^2^h^−1^) [20–28 kΩ]. As shown by Monteiro et al.^22^ the phase correlation length in our 2DES is about 170 nm, whereas the lithographically determined channel-width is about 150 nm. It is therefore reasonable to interpret the data from a quantum transport perspective. For low carrier densities the Fermi wavelength *λ*_F_ is several tens of nanometers^8^. This and the relatively low value of *g*_n_ suggest that we have only a few modes with a finite transmissivity in the channel. With increasing *V*_R_ we do not observe the expected quantum transport step-like features in *g*_n_, although the trace in Fig. 1c is obviously not monotonous. This is not surprising because for currents larger than *I*_c_ we approach the high bias regime where the conductance steps are known to quickly disappear^9,23^. In order to extract the transmissivity of the weak link we calculate from *I*_exc_ and *g*_n_ the barrier strength *Z* as a function of *V*_R_ using the BTK-formalism for an S–S interface^24^. *Z* is related to the normal state transmission probability *τ* by *τ* = (1 + *Z*^2^)^−1^. In this manner we obtain *Z* ≈ 0.8 and, correspondingly, *τ* ≈ 0.6 [Fig. 3e]. Comparison with the measured *g*_n_ thus suggests a total mode conductance of 2e^2^h^−1^, such that *g*(*τ* = 0.6) = 0.6 × 2e^2^h^−1^, close to the measured values. If we follow recent experiments by Gallagher et al.^9^ who observed e^2^h^−1^ modes in a normal state QPC, we could also consider only one mode with a higher transmissivity. However, this would require a re-analysis of the excess current based on an unconventional order parameter.

If we continue the discussion in the conventional picture, for a SQPC with perfect transmission (*τ* = 1) Beenakker and van Houten^3^ found that the critical current is given by *I*_c_ = *Ne*Δ(*ħ*)^−1^, where *N* was chosen to represent the number of spin degenerate modes (which contribute each 2*e*^2^*h*^−1^ to the normal conductance). Using this relation and including the obtained *τ* as a pre-factor, we can calculate the maximum supercurrent expected for our device, which yields *I*_c_ ≈ 3 nA. This is in good agreement with the measured *I*_P_, as can be seen in the bottom panel in Fig. 3e. For comparison we also plot the expected *I*_c_ for a diffusive junction^25^, which clearly gives much smaller values. The critical current *I*_c_ ≈ 3 nA implies a Josephson coupling energy *E*_J_ = 6.2 μeV. This is comparable to the bath temperature, *k*_B_*T*_base_ = 3.4 μeV. Therefore, as for the few-mode atomic scale point contacts^26,27^, the supercurrent is rounded.

### Zero-dimensional charge island with discrete states

**Fig. 4:**
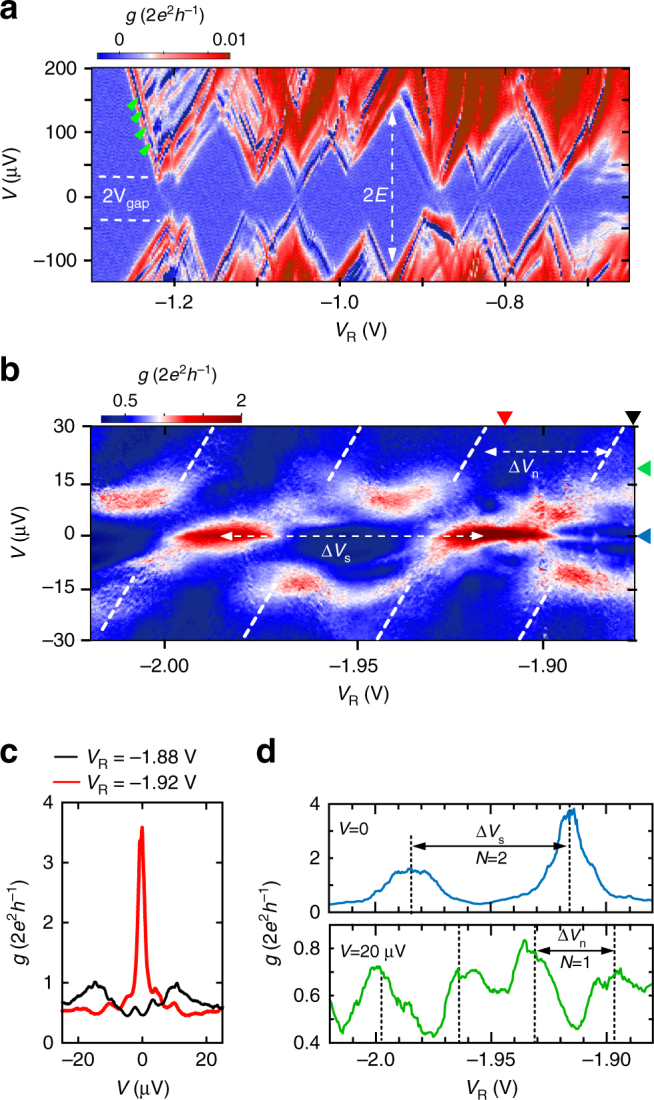
Charge island in regimes IV and III. **a** Conductance diamonds measured in regime IV with voltage bias *V* on the vertical axis. *E* denotes the addition energy of the island. The diamonds exhibit a gap of *V*_gap_ ≈ ±30 μV. Green arrows indicate signatures of excited states due to quantum confinement. Note that due to gate history effects, this regime occurs at a lower *V*_R_ range. **b** *g* measured in  regime III. For small voltages *g* exhibits peaks with periodicity Δ*V*_s_ = 70 mV. For *V* > 15 μV the periodicity increases by a factor 2, Δ*V*_n_ = 35 mV (dashed white lines). Black, red triangles and blue, green triangles indicate the line cuts shown in **c**, **d**, respectively. **c** vertical line cuts from **b** at *V*_R_ = −1.88 V and *V*_R_ = −1.92 V. **d** Horizontal line cuts from **b** at V = 0 (top panel) and *V* = 20 μV (bottom panel). The change in periodicity by a factor 2 suggests a change in number of transferred charges from *N* = 2 to 1, indicative for a superconducting island

Let us now turn to the regime of CDs, regime IV. Figure 4a presents a detailed measurement of *g* in this region. Note that this measurement was carried out in a voltage bias configuration. We observe a series of CDs whose size *E* on the (vertical) voltage axis is of the order of 80–150 μV. In gating experiments with non-superconducting materials, for instance in narrow semiconductor channels or graphene nano ribbons, CDs are known to occur in the low density limit, at the metal–insulator transition, because of puddles of charge carriers which form due to small inhomogeneities in the potential landscape, thus leading to quantum dot-like transport behavior^28–33^. A similar behaviour is also conjectured to occur in 2D superconductors around the transition from the superconducting to the insulating state^34,35^. From this analogy we infer that in regime IV the superfluid inside the constriction is at the transition to full depletion. Similar observations have been reported recently also by Prawiroatmodjo et al.^36^. The size of the CDs directly reflects the addition energy *E* that has to be paid in order to change the island occupation number and thus, to enable transport. *E* is composed of various contributions of which the most dominant ones typically are the Coulomb charging energy *U* = *Ne*^2^(2*C*_Σ_)^−1^ [with *C*_Σ_ being the total capacitance of the island and *N* the number of charges to be added or removed] and the energy level quantization due to quantum confinement *δε*. For quantum dots in LAO/STO^18,19,37^, Coulomb contributions are small because the STO substrate exhibits an extremely large dielectric constant $\epsilon _r$ = 25,000 at low *T* and for small electric fields^38^ which suppresses Coulomb repulsion. For our device, however, the fields originating from the split gates can not be neglected^22^. We have performed simulations of the dielectric environment in the region surrounding the constriction using finite element analysis (see Supplementary Note 2). Our results indicate that the geometry of the gates leads to a strong field focusing effect which reduces $\epsilon _r$ in the constriction such that Coulomb repulsion becomes relevant. The numerical simulations yield charging energies of *U* ≈ 100 μeV for an island with ~50 nm radius, compatible with our experiment. The data in Fig. 4a, further shows signatures of transport through excited states originating from quantum confinement, as can be seen from the fine structure of conductance lines parallel to the diamond edges between two adjacent diamonds [green arrows in Fig. 4a]^31,39^. This allows us to estimate *δε* ≈ 10–20 μeV, which would lead to an island size of ~80 nm, similar to the size obtained from the finite element simulations of the electrostatic properties. These values are also compatible with the electronic inhomogeneities typically observed in LAO/STO, which correlate with structural effects^15,40,41^.

The island couples to superconducting reservoirs, which can be inferred from the voltage gap *V*_gap_ ≈ ±30 μV that separates the CDs in positive and negative bias direction^42,43^. As expected, *V*_gap_ vanishes when a perpendicular magnetic field *B* = 1 T is applied (see Supplementary Note 4). We further observe pronounced negative differential conductance (NDC) along the edges of the CDs, which can be related to the sharp changes in density of states in the superconducting reservoirs around ±Δ. Since NDC occurs symmetrically for both positive and negative bias, we conclude that both reservoirs exhibit a superconducting energy gap (see Supplementary Note 5). When we compare the value of *V*_gap_ = 2Δ with the superconducting gap in the reservoirs, Δ ≈ 22 μeV, we obtain reasonable agreement. We note that in this regime IV the level spacing of quantum states on the island is of the same order as the superconducting gap, *δε* ~ Δ. We are therefore in the limit of Anderson’s criterion of superconductivity at small scales (*δε* < Δ)^44,45^.

### Charge island with superconducting ground state

Finally we turn to the strong coupling regime III [Fig. 4b]. The pattern of gapped CDs is not visible here. Instead we observe zero bias conductance peaks which are of the order of the quantum of conductance, *g* ≥ (2*e*^2^*h*^−1^), [cf. Fig. 4c, red curve]. They alternate with regions where *g* is suppressed. This suggests that the island is more transparent in this regime, allowing for Cooper pair transport^46^ at zero bias. The peaks in *g* occur periodically in Δ*V*_R_, with a periodicity Δ*V*_s_ = 70 mV [Fig. 4d, top panel]. Above a certain bias voltage *V* ≈ ±15 μV, the periodicity changes by a factor 2, Δ*V*_n_ = 35 mV [Fig. 4d, bottom panel]. This suggests that the parity of the island influences its energy state, as expected for a superconducting island^42^. In its ground state the island hosts Cooper pairs (even parity) and thus exhibits a charging energy 2*U*, reflecting the Cooper pair’s charge 2e (*N* = 2). Above a critical bias voltage the odd-parity state becomes available for quasi particles in the reservoirs thus enabling single electron transport across the island (*N* = 1). This results in period doubling of the Coulomb blockade oscillations. Our data therefore suggest that in the strong coupling regime III the island is in a superconducting state, thus forming a superconducting quantum dot.

## Discussion

We have realized a SQPC with split gate technology in a 2D superfluid. Because the superconducting point contact and the superconducting equilibrium reservoirs are made from one and the same material, transport becomes independent of unknown material interfaces, different Fermi velocities and atomic mismatch. Our system can serve as a unique experimental platform for future experiments on 2D superconductivity. This will make it possible to study, for example, the microscopic properties of the LAO/STO interface superconductivity, but also the properties of genuinely SQPCs as originally envisioned^3^. It may furthermore enable the investigation of nano scale superconductivity in few electron quantum dots.

## Methods

### Device fabrication

We use single crystal TiO_2_ terminated, (001) oriented SrTiO_3_ (Crystec ©GmBH) as a substrate without further modification. The fabrication involves three electron beam lithography steps (EBL), which are carried out using a double layer resist (PMMA 495K/950K, thickness 100/200 nm, baked for 15 min at 175 °C) which is exposed with a dose of typically 800–900 μCcm^−2^ and developed using a MIBK:IPA, 1:3 solution (90 s). The first EBL step defines the positions of reference markers which are obtained by sputtering 60 nm Tungsten (*W*) at pressure *p* = 0.02 mbar and consecutive ultrasonic lift-off. The second EBL step patterns the geometry of the device: Those regions which are to remain insulating are covered with 20 nm of sputtered AlO_2_ (*p* = 0.003 mbar, 200 W power, 20 sccm Ar flow; lift-off process in 50 °C acetone). Next, the LAO layer is grown by means of pulsed laser deposition (PLD) at 770 °C with an O_2_ pressure of *p*_O2_ = 6 × 10^−5^ mbar. Only in those regions which are not covered by the AlO_2_ hard mask growth is crystalline such that the STO surface is covered with a 12 unit cell (5 nm) LAO layer, giving rise to the 2DES at the interface. In all other regions the AlO_2_ mask prevents the formation of the 2DES and the LAO layer is amorphous. Growth is monitored in situ by reflection high energy electron diffraction which confirms layer-by-layer growth. After LAO deposition, the sample is annealed for 1 h at 600 °C and at a pressure of *p*_O2_ = 300 mbar in order to suppress the formation of O_2_ vacancies. The final EBL step defines the pattern of gate electrodes. Polymer residuals are removed with an Oxygen plasma (15 s, 200 W, 212 sccm *O*_2_ flow). The metal layer for the surface gates is deposited using electron beam evaporation (*p*_base_ ≤ 5 × 10^−8^ mbar). This layer consists of 100 nm Au. No sticking layer is used. The first 20 nm Au are deposited at a rate of 0.5 Å s^−1^. Then the rate is increased to 1 Å s^−1^ until the final thickness of 100 nm is reached. Using a syringe for the lift-off with 55 °C acetone ensures a gentle procedure that prevents the gates from peeling off. The sample is then mounted in a chip carrier with silver paint, serving as a back gate. Ultrasonic wedge bonding provides Ohmic contacts to the 2DES.

### Electrical measurement setup and device characterization

All measurements (unless stated otherwise) are performed using dc electronics, with the current sourced at reservoir S of the sample and drained at reservoir D. The resulting voltage drop *V* is probed at separate contacts in the respective reservoirs. The dilution refrigerator is equipped with copper powder filters, which are thermalized at the mixing chamber, and Pi-filters at room temperature. The carrier density in the 2DES is adjusted globally by applying a negative back gate voltage *V*_BG_ = −1.875 V, which corresponds to a reduced density compared to *V*_BG_ = 0. We determine the carrier density from Hall measurements performed at 300 mK using voltage probes on opposite sides of the reservoir with width *w* = 150 μm. The longitudinal resistance is determined from voltage measurements between probes separated by l = 112.5 μm. This yields a carrier density *n* ≈ 3 × 10^13^ cm^−2^ and a mobility *μ* ≈ 800 cm^2^ V s^−1^. For this carrier density we observe the resistively measured superconducting transition at *T*_c_ ≈ 100 mK, which corresponds to a BCS gap Δ_Tc_ = 15 μeV.

### Data availability

The data that support the findings of this study are available from the corresponding author upon reasonable request.

## Electronic supplementary material

Supplementary Information

Peer Review File
