# Supplementary material for: Transport regimes of a split gate superconducting quantum point contact in the two-dimensional LaAlO3/SrTiO3 superfluid
Source: Nat Commun. 2018 Jun 11;9:2276. doi: 10.1038/s41467-018-04657-z (PMC5995834; doi:10.1038/s41467-018-04657-z)
Supplement: Supplementary file 1 — Supplementary Information [file 41467_2018_4657_MOESM1_ESM.pdf]

Supplementary Information: Transport regimes of a split gate superconducting quantum point contact in the two-dimensional $LaAlO_3/SrTiO_3$ superfluid

Thierschmann *et al.*

Supplementary Table I: Detailed Information on the Device Fabrication

Step	
	**First electron beam lithography (EBL) step: Tungsten (W) markers.**
1.	Single crystal TiO_2 terminated, (001) oriented $SrTiO_3$ substrate (Crystec © GmBH)
2.	2-layer resist spinning: PMMA 495K/950K, 4000 rpm, thickness 100/200 nm.
3.	Bake at 175°C for 15 min.
4.	Spinning of conductive polymer (Elektra92 (AR-PC-5090)): 4000 rpm, 60 nm.
5.	Bake at 110 °C, 1 min.
6.	Ebeam exposure: 850 $\mu C/\mathrm{cm}^2$.
7.	Dissolve Elektra92 in DI water: 2 min. at 40°C.
8.	Develop in MIBK:IPA solution (1:3) for 90 sec.
9.	Rinse with IPA.
10.	RF-sputtering of W: thickness: 60nm, pressure p=0.02 mbar.
11.	Lift-off in ultrasonic Acetone bath (55 °C).
	**Second EBL step: Patterning of the insulating regions using a AlO_2 hard mask.**
12.	2-layer resist spinning: PMMA 495K/950K, 4000 rpm, thickness 100/200 nm.
13.	Bake at 175°C for 15 min.
14.	Spinning of conductive polymer (Elektra92 (AR-PC-5090)): 4000 rpm, 60 nm.
15.	Bake at 110 °C, 1 min.
16.	Ebeam exposure: 850 $\mu C/\mathrm{cm}^2$.
17.	Dissolve Elektra92 in DI water: 2 min. at 40°C.
18.	Develop in MIBK:IPA solution (1:3) for 90 sec.
19.	Rinse with IPA.
20.	Sputtering of AlO_2: 20 nm, p= 0.003 mbar, 200 W, 20 sccm Ar flow.
21.	Lift-off in ultrasonic Acetone bath (55 °C).

	**Growth of the $LaAlO_3$ layer with Pulsed Laser Deposition.**
22.	Mount sample, heat up to T=770 °C.
23.	Start growth: O_2 pressure: 6×10^{-5} mbar, laser fluency 1J/cm^2, repetition rate 1Hz.
24.	Monitor thickness (number of layers) in RHEED.
25.	Stop growth of after 12 u.c.
26.	Increase O_2 pressure to 300 mbar.
27.	Decrease T to 600 °C.
28.	Anneal for 1h.
29.	Cool down to room temperature inside the chamber.
30.	Establish ambient conditions.
	**Third EBL step: patterning of the gate electrodes.**
31.	2-layer resist spinning: PMMA 495K/950K, 4000 rpm, thickness 100/200 nm.
32.	Bake at 175°C for 15 min.
33.	Spinning of conductive polymer (Elektra92 (AR-PC-5090)): 4000 rpm, 60 nm.
34.	Bake at 110 °C, 1 min.
35.	Ebeam exposure: 850 μC/cm^2.
36.	Dissolve Elektra92 in DI water: 2 min. at 40°C.
37.	Develop in MIBK:IPA solution (1:3) for 90 sec.
38.	Rinse with IPA.
39.	Remove polymer residues with oxygen plasma: 200 W, 212 sccm O2 flow for 15 sec.
40.	Ebeam evaporation of 100 nm Au: $p < 5\ 10^{-8}$ mbar
40.a	Deposit 20 nm Au with rate 0.5 Å/s nm
40.b	Deposit 80 nm Au with rate 1 Å/s nm
41.	Lift-off: rinse the sample with warm Acetone (55 °C) using a syringe.
42.	After processing the chip is glued into a chip carrier with silver paint, which also serves as a back gate.
43.	Ohmic contacts are provided though ultrasonic wedge bonding.

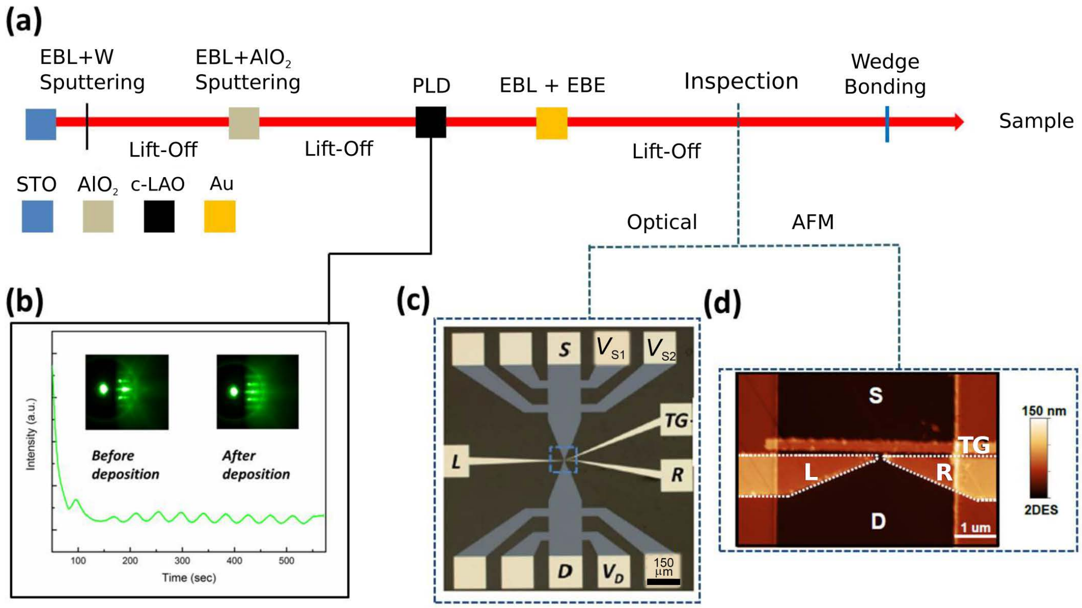

Supplementary Figure 1. **Device fabrication (a)** Fabrication flow. The color legend indicates the deposited materials at the different stages. Note that along with the gates (Au) we also pattern Au-squares as bond pads for Ohmic contacts. These Au pads only serve as markers to enhance the visibility during bonding. **(b)** The in-situ RHEED oscillations monitored during growth. They confirm layer-by-layer growth. The insets show the RHEED diffraction pattern before and after the deposition of the LAO layers. **(c)** Optical image of the device after the Au-lift off process. Ohmic contacts and the gate electrodes are labelled. **(d)** Atomic Force Microscopy (AFM) image showing the top gate architecture of the device.

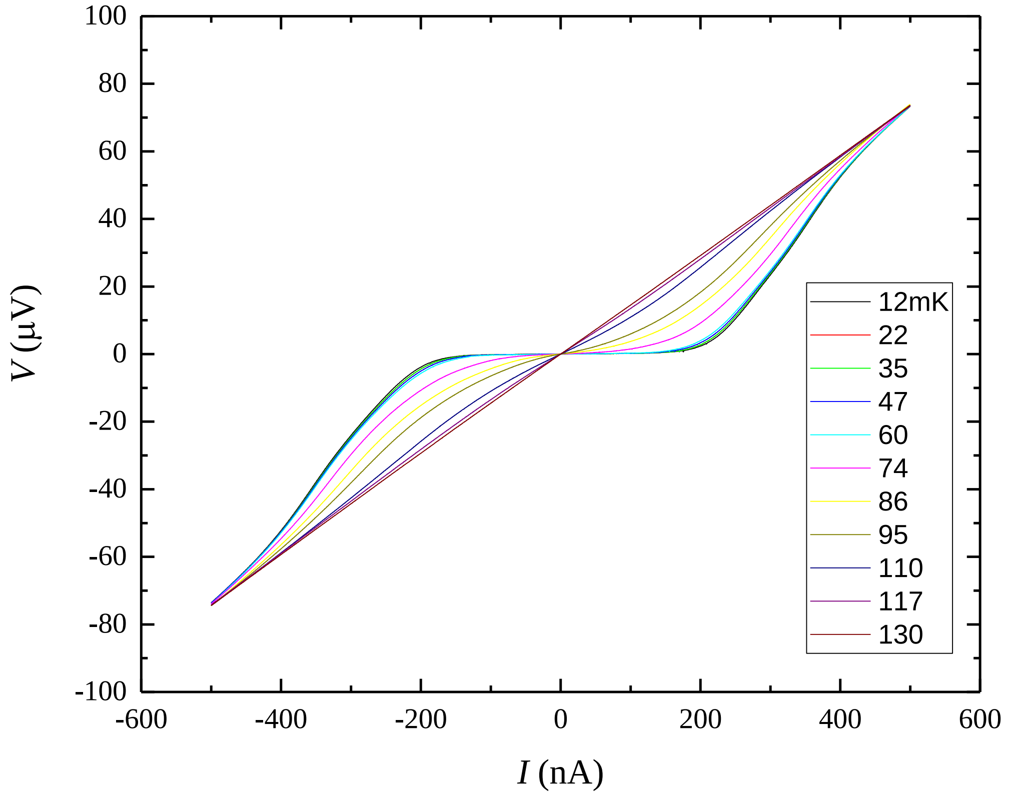

Supplementary Figure 2. **Voltage-current curves for various temperatures** measured in the reservoirs of the device using contacts S and D as source and drain for the current and V_{S1} and V_{S2} to probe the resulting voltage drop V. We obtain $T_c \approx 100$ mK if we use $R_{Tc} = R(130 \text{ mK})/2$ to define T_c. Using the BCS equation for the superconducting gap Δ, this yields $\Delta = 1.76 k_B T_c \approx 15\ \mu$eV

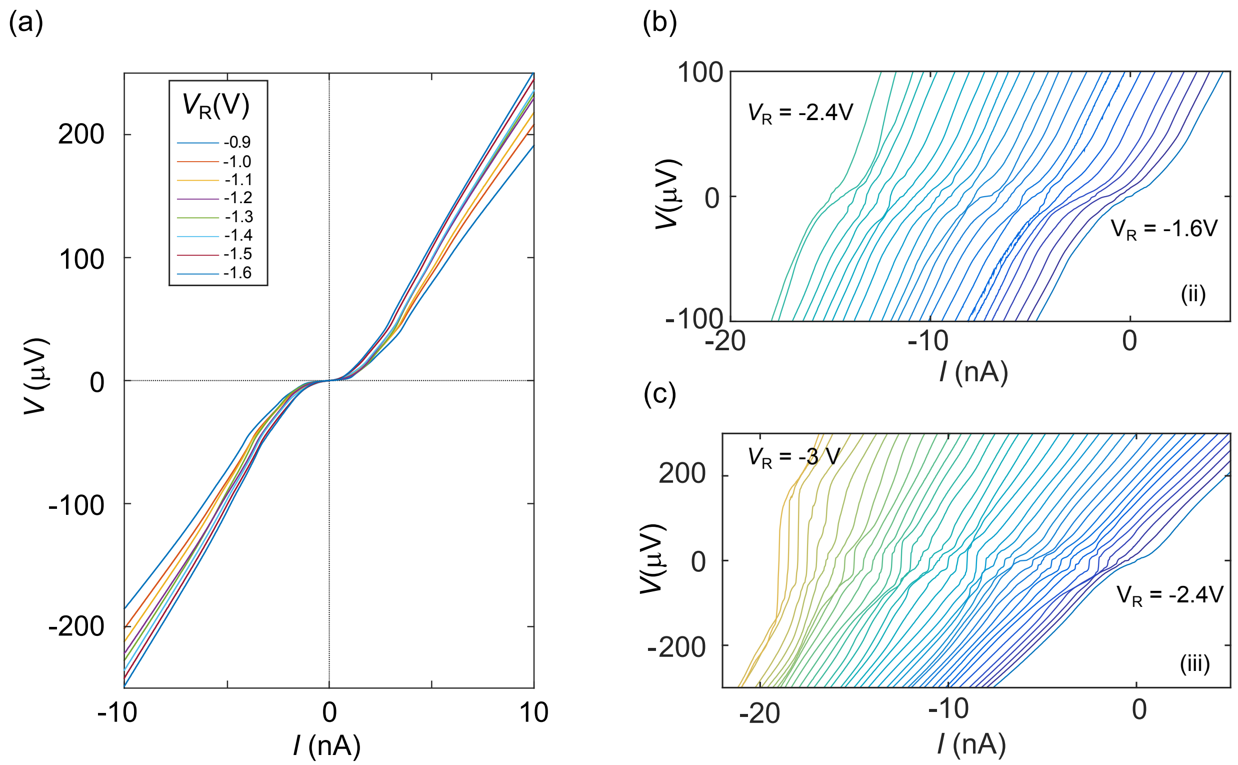

Supplementary Figure 3. ***I*-*V* curves for Regimes II to IV (a)** Regime II (V_R = -0.9 to -1.6 V in steps of 0.1 V.) **(b)** Regime III (V_R=-1.6 V to -2.4 V in steps of 32 mV). The curves are offset along the I-axis by -0.6 nA. **(c)** Regime IV ($V_R = -2.4$V to -3V in steps of 16 mV). The curves are offset along the I-axis by -0.5 nA.

SUPPLEMENTARY NOTE 1: CALCULATION OF THE BARRIER TRANSPARENCY AND THE CRITICAL CURRENT IN THE WEAK LINK REGIME II

We use the Blonder-Tinkham-Klapwjik (BTK) formalism described in Ref. [1] to calculate the barrier parameter Z from the excess current $I_{\rm exc}$ and the high bias conductance $g_{\rm n}$. $I_{\rm exc}$ at an S-S interface is related to the Z parameter by

$$I_{\rm exc} = 2\frac{g_{\rm n}}{e(1-B(\infty))} \times \int_0^\infty dE(A(E) - B(E) + B(\infty)), \tag{1}$$

with

$$A = \frac{\Delta^2}{E^2+(\Delta^2-E^2)(1+2Z^2)^2}, \tag{2}$$

$$B = 1 - A, \tag{3}$$

for $E < \Delta$, and

$$A = \frac{u_0^2 v_0^2}{\gamma^2}, \tag{4}$$

$$B = \frac{(u_0^2-v_0^2)^2 Z^2 (1+Z^2)}{\gamma^2}, \tag{5}$$

for $E > \Delta$. Furthermore, $B(\infty) = \frac{Z^2}{1+Z^2}$, and

$$u_0 = \tfrac{1}{2}(1 + ((E^2 - \Delta^2)/E^2)^{1/2}, \tag{6}$$

$$v_0 = 1 - u_0^2, \tag{7}$$

$$\gamma = (u_0^2 + Z^2(u_0^2 - v_0^2))^2). \tag{8}$$

We determine $I_{\rm exc}$ from the experimental data by extrapolating a linear fit to high bias conductance $g_{\rm n}$ towards V=0. This yields the data shown in the top panel of Fig. 4(e) in the main text (with a relative error < 0.01). Combining this with the respective $g_{\rm n}$ for each gate voltage and using Δ=22 μeV (with relative error $\sim$ 0.1), as extracted from the dI/dV vs V curves (Fig. 3(c) in the main text), allows us to calculate the corresponding Z parameter, which leads to the curve shown in Supplementary Figure 4. By comparison we can then determine the Z parameter as a function of $V_{\rm R}$ and calculate the corresponding transmission coefficient in the normal state, $\tau = 1/(1 + Z^2)$ [1].

The critical current of a SQPC with N with perfectly transmitting ($\tau = 1$) channels with $2e^2{\rm h}^{-1}$ conductance each as provided by Beenakker and van Houten [2] is given by

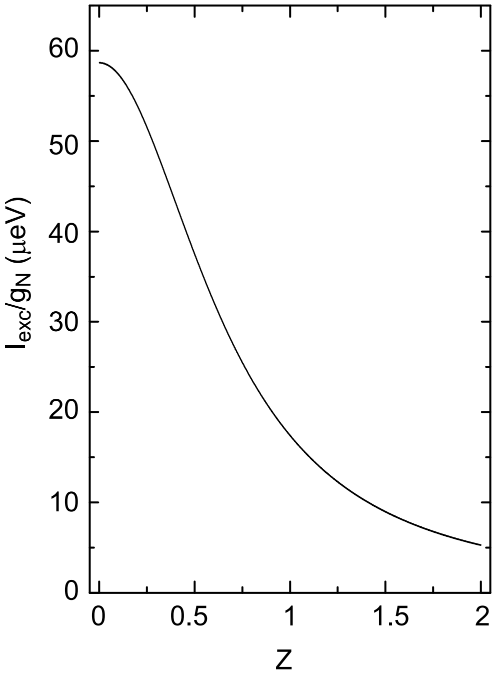

Supplementary Figure 4. **Calculated Z-parameter** Experimentally determined $I_{\mathrm{exc}}/g_{\mathrm{n}}$ and the corresponding Z-parameter determined for an S-S interface using BTK theory.

$I_{\mathrm{c}} = Ne\Delta/\hbar$. Combining this expression with the gate voltage dependent τ and using $N = 1$ yields the red dashed line in the bottom panel in Fig. 3(e).

For the critical current I_{c} of a point contact in the diffusive transport regime we apply the equation provided by Beenakker in Ref. [3]

$$I_{\mathrm{c}} = 1.32\frac{\pi\Delta}{2e}\langle G\rangle \tag{9}$$

where we have used the experimentally determined g_{n} for the average conductance $\langle G\rangle$ and $\Delta = 22$ μeV. This yields the blue dashed curve shown in the bottom panel in Fig.4(e) in the main text.

SUPPLEMENTARY NOTE 2: NUMERICAL SIMULATIONS OF THE ELECTROSTATIC ENVIRONMENT IN THE CONSTRICTION

We model the dielectric environment of the constriction by finite elements analysis. For that purpose we developed a 3D model of our device in COMSOL®5.2. The simulation is developed similarly to that reported by Monteiro *et al.* [4], based on the electrostatic module. The structure is modeled with 3 layers stacked on top of each other (Supplementary Figure 5(a)). At the top there is a 5 nm-thick $LaAlO_3$ layer, having a dielectric constant of 24 [5] and insulating character. The middle layer is the 2DES, modelled as a 10-nm thick metal with conductivity calculated from the experimental data. The bottom layer is 1 μm-thick $SrTiO_3$, which is an insulator with a field-dependent dielectric constant described by the Landau-Ginsburg-Devenshire Theory [6, 7]:

$$\varepsilon_{\rm STO} = 1 + \frac{B}{[1 + (E/E_0)^2]^{2/3}}, \tag{10}$$

with E being the local electric field, $B = 25000$ and $E_0 = 82000$ V/m [8]. The split gates L and R are modelled as 100 nm thick triangular Au electrodes. The tips of the split gate are separated from each other by the distance $D = 150$ nm, corresponding to the nominal distance between the split gates of the device discussed in the main text. Gate voltages V_L and $V_{\rm R}$ are applied to the respective gates with respect to the drain reservoir, which is kept at ground potential. The source reservoir is voltage biased. The island is modelled as a conductive disc with diameter d, which is separated from source and drain by gaps of width g.

Supplementary Figure 5(b) shows the spatial map of $\varepsilon_{\rm r}$ at the LAO/STO interface for $V_{\rm L} = -1$V and $V_{\rm R} = 0$. This visualizes the huge $\varepsilon_{\rm r}$ in large parts of the sample. In Supplementary Figure 5(c) the $\varepsilon_{\rm r}$ -map is shown for both gates at the same voltage ($V_{\rm L} = V_{\rm R} = -1$V). This results in a strong reduction of the dielectric number by more than one order of magnitude in the constriction and thus in the vicinity of the island.

The single electron charging energy of the island $E_{\rm C}$ is extracted by calculating the voltage between source and drain that is required to change the polarization on the island by e (electronic charge) while $V_{\rm L,R} = -1$V. This calculation is carried out for different values of the island's diameter d and the gap g. The result is shown in Supplementary Figure 6(a). Charging energies of 60 μeV [marked by the dashed line in Supplementary Figure 6(a)] to

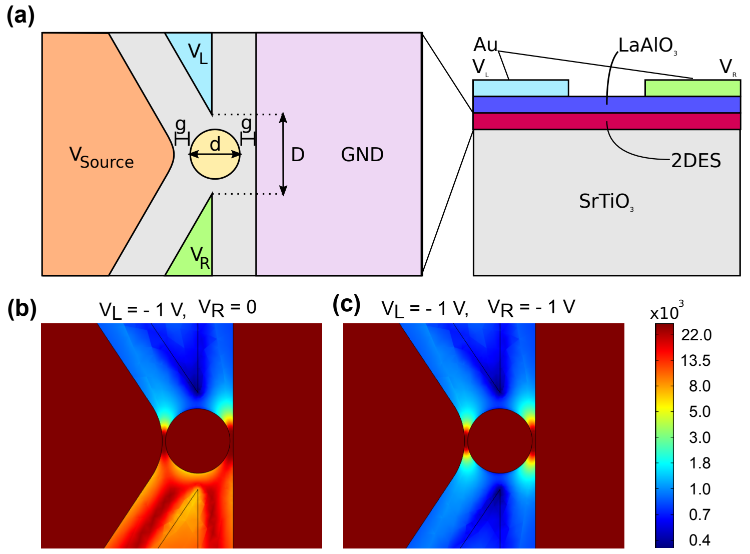

Supplementary Figure 5. **Finite element analysis model of the device (a)** Layer stack and geometry. **(b)** spacial map of the dielectric constant in the constriction at low temperature for gate voltages $V_{\mathrm{L}} = -1\mathrm{V}$, $V_{\mathrm{R}} = 0\mathrm{V}$ and **(c)** for $V_{\mathrm{L}} = -1\mathrm{V}$, $V_{\mathrm{R}} = -1\mathrm{V}$.

100 μeV are obtained for island diameters of 70 nm to 120 nm if one allows g to vary between approximately 3 nm and 6 nm. For comparison we also show the charging energy of the island for the case when the electric field dependence of $\varepsilon_{\mathrm{STO}}$ given by eq. 10 is neglected (open circles). Clearly the obtained charging energies are an order of magnitude smaller, emphasizing the relevance of eq. 10 for split gate devices in LAO/STO.

SUPPLEMENTARY NOTE 3: ANALYSIS OF EXCITED STATES SIGNATURES

We can further use the transport signatures of (excited) quantum states observed in the conductance diamond regime IV to estimate the size of the island.

A close up of the voltage range where these features are observed is depicted in Supplementary Figure 6(b). Only positive bias voltages are shown. The delimiting lines of the Coulomb diamonds are indicated with blue lines. Transport signatures of excited states of the island due to quantum confinement can be observed in the region between two adjacent Coulomb diamonds. They appear as lines of enhanced conductance which run in parallel with the borders of the Coulomb diamonds [9, 10]. In Supplementary Figure 6(b) they are denoted with green lines. The separation between these lines along the (vertical) bias

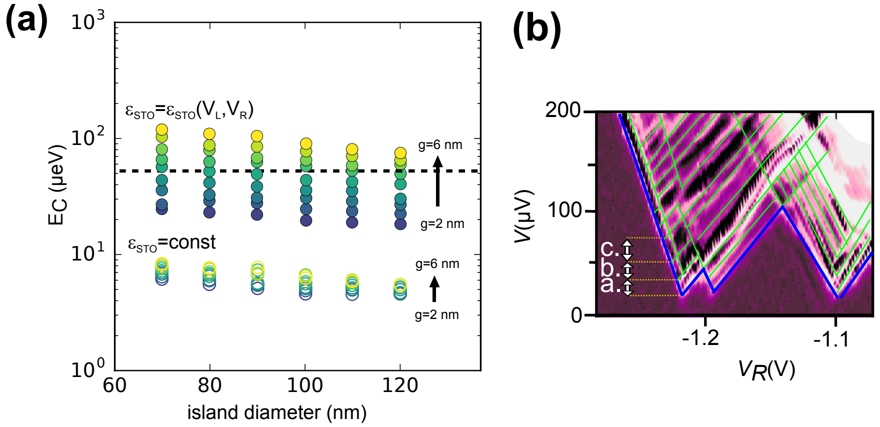

Supplementary Figure 6. **Analysis of the Coulomb Diamonds (a)** Results of the numerical simulation for $V_{\mathrm{L}} = -1\mathrm{V}$, $V_{\mathrm{R}} = -1\mathrm{V}$. The single electron charging energy is plotted as a function of island diameter. Colors indicate the assumed gap size ranging from $g = 2$ to 6 nm. Solid circles show the results obtained when the electric field dependence of ϵ_{STO} is taken into account. Open circles correspond to the case when this effect is neglected. **(b)** Close-up of the conductive region between two adjacent Coulomb diamonds, extracted from the main text. The borders of the diamonds are denoted with blue. Green lines indicate transport signatures of excited states on the island due to quantum confinement. The energy separation between different states are indicated with $a = 14\ \mu\mathrm{V}$, $b = 17\ \mu\mathrm{eV}$, $c = 21\ \mu\mathrm{eV}$.

voltage axis indicates their difference in energy, $\delta\varepsilon$. As an example, the energy separation of 4 such lines is indicated in Supplementary Figure 6(b) with $a = 14\ \mu\mathrm{eV}$, $b = 17\ \mu\mathrm{eV}$, $c = 21\ \mu\mathrm{eV}$. Using a simple particle-in-a-box picture, we can estimate the spatial dimension d required to obtain quantization energies of this order,

$$d = \sqrt{\frac{h^2}{8m\delta\varepsilon}}, \tag{11}$$

where $m = 0.7m_{\mathrm{e}}$ is the effect electron mass in the LAO/STO 2DES, m_{e} is the the bare electron mass and h is Planck's constant. Approximating by using $\delta\varepsilon = 20\mu eV$ yields an island radius of approximately 80 nm. This is in the same range as the result obtained from the purely electrostatic considerations above.

This analysis clearly shows that the energy scale of the conductance diamonds is at least a factor 3 larger than that observed for the electronic orbital contributions originating from

quantum confinement. Moreover, the numerical simulations clearly show that Coulomb repulsion cannot be neglected in the device. The results strongly suggests that Coulomb blockade is the main contribution to the observed conductance diamonds. Using both signatures to estimate island size independently yields consistent results.

SUPPLEMENTARY NOTE 4: COULOMB DIAMOND REGIME IV FOR LARGE MAGNETIC FIELD

Supplementary Figure 7 (a) shows the series of Coulomb diamonds discussed in the main text with a perpendicular magnetic field B=1 T applied. Since B is much larger than the typical critical magnetic field in superconducting LAO/STO 2DES, $B_c \approx 0.2$T, superconducting transport in the leads is suppressed. Therefore, the voltage gap $V_{gap} \approx 30\ \mu$eV observed for $B = 0$ vanishes. The zero bias conductance is given in Supplementary Figure 7(b). Interestingly, the amplitudes appear to alternate in an odd-even manner, resembling the parity effect in superconducting islands with two normal electrodes due to quasi-particle poising [11] or photon assisted tunneling processes from radiation leaking though an imperfect shielding [12]. This could suggest that despite the high magnetic field paired electrons are still present on the island. In the light of recent publications [13–15] this could hint at another signature of electron pairing without macroscopic superconductivity in LAO/STO.

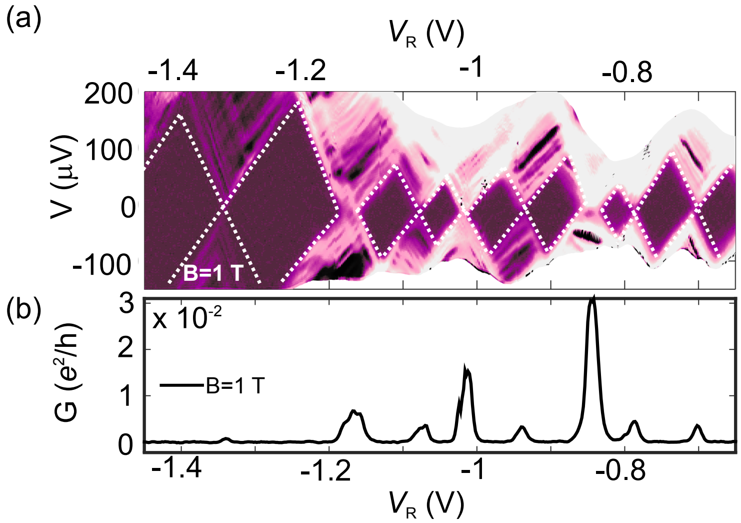

Supplementary Figure 7. **Coulomb diamonds in a magnetic field (a)** Coulomb diamonds discussed in the main text with a perpendicular magnetic field B=1T applied. It can be seen that the gap V_{gap} has vanished. **(b)** Zero bias conductance as a function of V_R extracted from (a).

SUPPLEMENTARY NOTE 5: NEGATIVE DIFFERENTIAL RESISTANCE AND SUB GAP FEATURES IN THE COULOMB DIAMOND REGIME IV

We observe clear signatures of negative differential conductance (NDC) at the edges of the Coulomb diamonds. They occur for both positive and negative bias voltage, which indicates the presence of a superconducting gap in both reservoirs. As an example, a close up of the X shaped structure observed around $V_{\mathrm{R}} = -0.75$ V is presented in Supplementary Figure8(a). Similar features have been observed recently by Cheng et al [14], who pointed out a connection to tunable electron-electron interactions on LAO/STO quantum dots. It can clearly be seen from Supplementary Figure 8(a) that the four 'arms' of the X intersect around $V = 0$ (◯). This suggests that conductance originates from alignment of the island state with states close to the Fermi levels in the reservoirs [cf. the corresponding energy diagram in Supplementary Figure 8(c)]. Each of the 'arms' exhibits a pronounced negative differential conductance (NDC). This becomes highlighted in Supplementary Figure8(b) where traces of g are shown that are obtained from the vertical line cuts denoted α and β in Supplementary Figure8(a). When going from $V = 0$ towards positive or negative bias, g exhibits first a positive peak at the intersections with the X structure, followed by a change of sign and a subsequent negative signal of approximately equal magnitude [black arrows in Supplementary Figure8(b)].

Generally, NDC reflects the alignment of the island state with a sharp DOS peak in the reservoirs. This becomes clear when we consider the energy diagrams presented in Supplementary Figure8(d) which sketch the configurations indicated with △ and □ in Supplementary Figure 8(a). For both configurations increasing the bias voltage misaligns the island state with the DOS peak in the reservoir and thus reduces the current, leading to NDC. From the symmetric occurrence of NDC with respect to both V and V_{R} we infer that both reservoirs exhibit a sharp DOS peak. The location of the peaks close to the Fermi level could indicate that it is related to the Cooper pair DOS. Recent tunneling experiments, on the other hand, show indications of quasi particle sub gap states [16], which might give rise to features similar to the ones observed here. We note that NDC at the boundaries of the Coulomb diamonds disappears if a magnetic field is applied, cf. Supplementary Figure7. This further confirms that the NDC observed here originates from superconductivity.

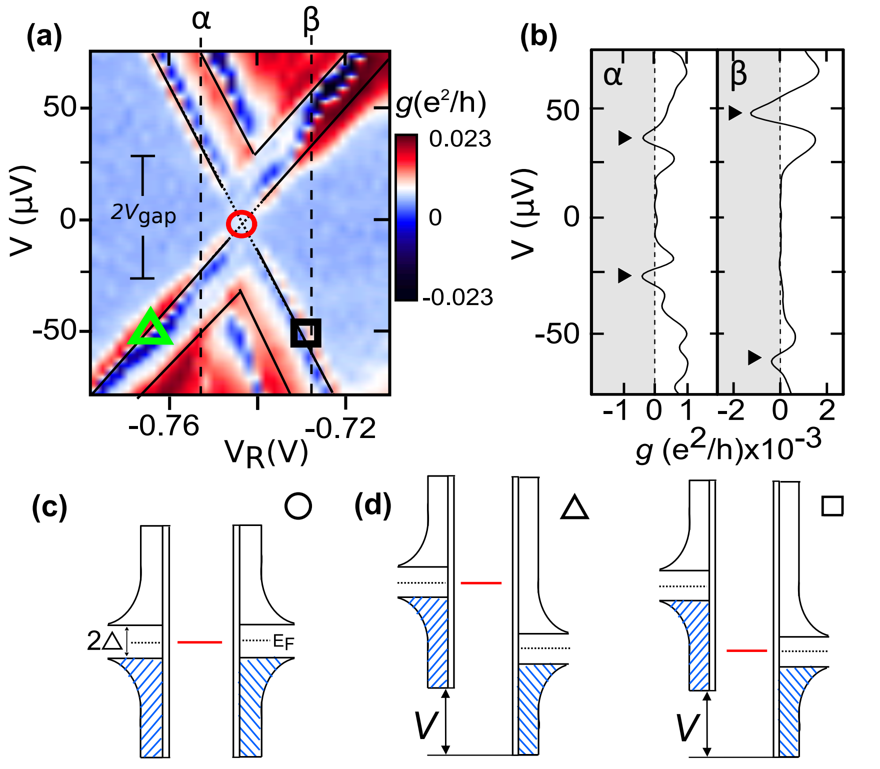

Supplementary Figure 8. **Negative differential resistance in the Coulomb diamonds of regime IV (a)** a close-up from Fig. 4a) in the main text around $V_{\mathrm{R}} = -0.75$ V. α and β denote vertical line cuts shown in (b) which highlight the NDC features occurring symmetric with respect to bias voltage. The symbols (◯, □,△) indicate the energy level configurations sketched in (c) and (d).

SUPPLEMENTARY NOTE 6: PRE-CHARACTERIZATION OF THE SPLIT GATES

Supplementary Figure 9 (a) shows measurements of the gate leakage currents I_{leak} measured at $T \sim 1$ K by recording the dc drain current while varying the voltage applied to the respective gate. We find that even at $V_{\mathrm{L,R}} = -4$ V the leakage current remains small, $|I_{\mathrm{leak}}| < 10$ pA, even at -4 V.

In order to characterize the influence of the gate, we bias the 2DES with a constant voltage (V=1 mV) and measure the drain current (I_{D}) while changing the applied gate voltage. Note that this was done at finite back gate voltage to reduce the global carrier density such that the insulating state (pinch-off) is reached within the available gate voltage

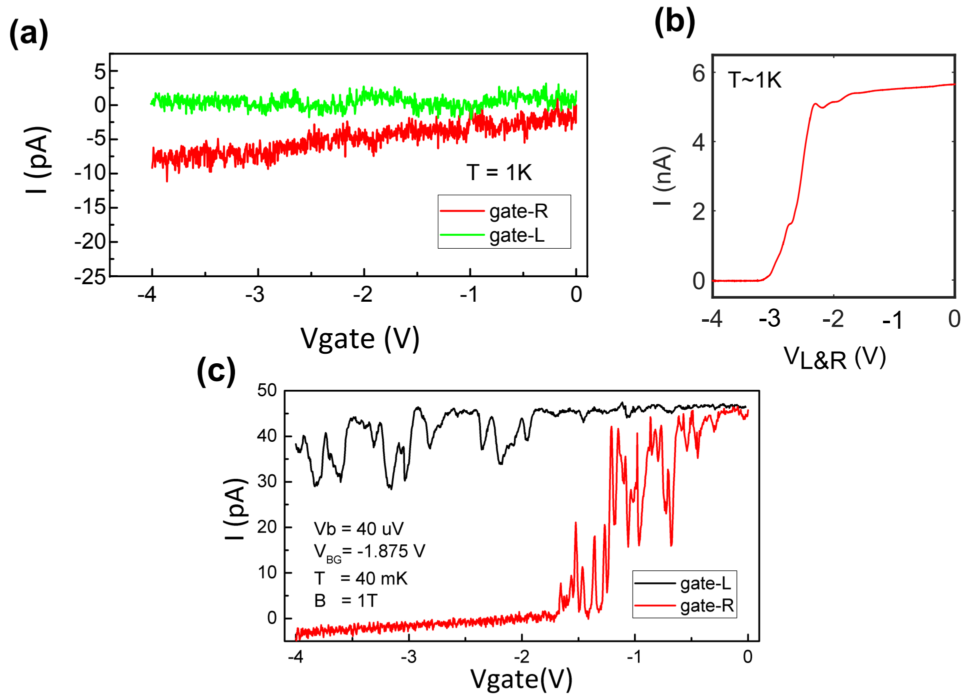

Supplementary Figure 9. **Pre-characterization of the split gates** (a) Leakage currents of the gates measured at $T = 1$K. (b) Pinch off curve at 1K for both gates L and R tuned simultaneously at 1K. **(c)** Pinch off curves when a voltage is applied only to one of the gates while the respective other one is grounded.

range. Supplementary Figure 9 (b) shows the result obtained for applying a voltage to gates L and R simultaneously. At low temperature, we observe the behaviour shown in Supplementary Figure 9(c). Varying gate R alone enables us to block transport through the channel. Gate L, in contrast, does not pinch off the current, even at $V_{\mathrm{L}} = -4V$. This indicates that the current does not flow through the 2DES underneath gate L, even at V_{L}=0, suggesting that it is depleted already at this stage and hence, the weak effect of gate L on transport. Note that superconductivity is suppressed in these measurements due to a perpendicular magnetic field $B = 1$T.

SUPPLEMENTARY NOTE 7: MEASUREMENTS ON DEVICE 2

Supplementary Figure 10 presents data on regime II obtained from another sample (device 2). An optical image and an atomic force microscope image of this device are shown in the top and bottom panel of Supplementary Figure 10(d), respectively. Note that, in contrast to the device discussed in the main text, this sample did not contain an additional, thin gate spanning the full LAO/STO channel. No back gate voltage was applied which is expected to result in a slightly higher carrier density in the 2DES and, consequently, in a larger superconducting gap than for the device in the main text.

Supplementary Figure 10(a) shows the differential resistance $r = \mathrm{d}V/\mathrm{d}I$ as a function of bias current I and gate voltage $V_{\mathrm{L+R}}$ in the range from -4 V to -6 V, where the SQPC is formed. The inset depicts a larger gate voltage range. Here we see that after the initial formation of the constriction ($V_{\mathrm{L+R}} \sim -0.5$ V) the gate voltages have to be swept over a larger range to reach regime II. This is different from the device in the main text and it is consistent with having a higher carrier density in the 2DES. In Supplementary Figure10 we have included this gate voltage range into regime I. Figs.10 (b), (c) and (e) - (h) depict the results of the SQPC analysis carried out as for the main text. We find a significant excess current (e), which can be used to calculate the junction transparency Z and the quasi particle reflection coefficient τ (f). If we use these parameters to calculate the normal state conductance g and the critical current I_{c} of the SQPC we yield good agreement with the measured conductance (g) and I_{P} (h) if a gap $\Delta = 27$ μeV and a total mode conductance $4e^2\mathrm{h}^{-1}$ are assumed.

We point out that I_p appears to be larger in this device compared to the sample discussed in the main text, which suggests that also the critical current and the Josephson coupling energy of the junction is lager. We also observe that at the onset of regime II, $V_{\mathrm{L+R}} \sim -4.5$ V, for small currents the I-V is almost flat (b), with a resistance of only a few 100 Ω (c), in contrast to the device in the main text. These two observations are linked as follows: As the Josephson coupling energy is larger in this device (while the bath temperature remains constant), thermal fluctuations are less likely to cause phase slip events, associated with the rounded supercurrent. Hence the lower resistance measured for smaller current in device 2. As the gate voltages are changed towards more negative values, the Josephson coupling energy gets smaller because the transmission of the quantum point contact is reduced. Hence,

the probability for thermally induced phase slips becomes enhanced and we observe an increasingly rounded supercurrent when the gate voltages are reduced. This nicely shows how the split gates affect the transmission through the constriction electrostatically.

The transitions to regime III and IV were not observed in this measurement. This is most likely related to the enhanced carrier density in the sample due the absence of a back gate voltage. As a result, larger values for the top gate voltages are necessary to access the same local carrier density and thus to tune the junction into the low carrier density regime. Applying even larger negative gate voltage values was not possible because for $V_{\mathrm{L+R}} < -6$ V leakage currents from the gates can typically not be neglected anymore.

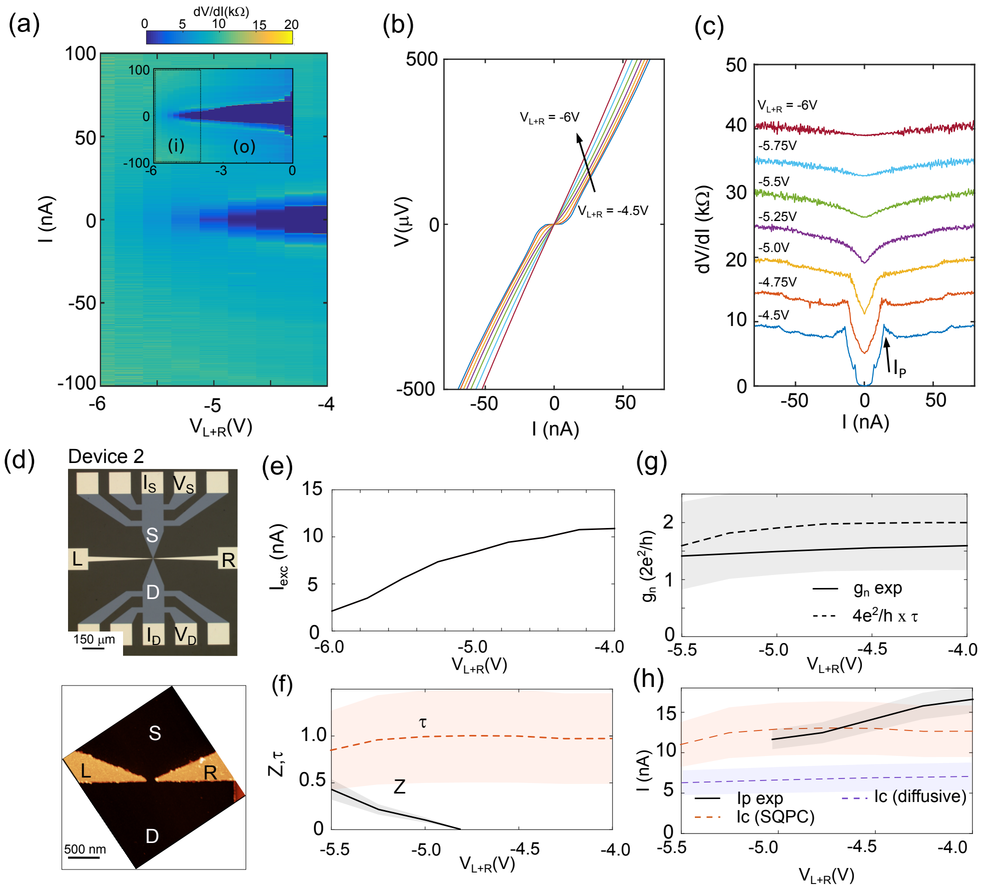

Supplementary Figure 10. **SQPC regime in Device 2 (a)** Differential resistance r as a function of current I and gate voltage $V_{\mathrm{L+R}}$ applied to left and right split gate at the same time. **(b)** $I-V$ curves for different gate voltages. **(c)** Differential resistance $r = dV/dI$ as a function of current I for different gate voltages $V_{\mathrm{L+R}}$. **(d)** Optical (top) and atomic force microscope (AFM) image of device 2, which contained only two wedge shaped split gates. **(e)** Excess current I_{exc} of the weak link determined from the I-Vs in (b). **(f)** Z parameter and τ determined from the I_{exc} as described in section , using $\Delta = 27 \pm 7\ \mu eV$. **(g)** Measured high bias differential conductance (g_n) compared to conductance expected from the calculated electron transmissivity τ assuming 2 conducting channels (N=2) contributing $2e^2\mathrm{h}^{-1}$ each. **(h)** Expected critical current I_c for a SQPC with N=2, $\Delta = 27\ \mu eV$ and τ from (f) [red, dashed line], compared with I_{P} extracted from the experimental data (black) and the supercurrent expected for a diffusive contact (blue dashes). Note that beyond $V_{\mathrm{L+R}} = -5V$ no I_{P} could be measured (cf. (c)).

Supplementary Figure 11 and Supplementary Figure 12 present results from a gate sweep obtained from device 2 showing a behavior as observed for regime III and IV in Figure 2 in the main text. These data were obtained from a different series of measurements than those discussed above (Supplementary Figure 10).

The characteristic signatures of regime III are observed in the gate voltage range from $V_{\mathrm{L}} = -2$ V to -2.85 V. In Supplementary Figure 11, which depicts the differential resistance $r = \mathrm{d}V/\mathrm{d}I$ as a function of gate voltage and bias current (cf. Fig 2(a) in the main text), we observe the alternating behavior of high and low resistance at small bias. Supplementary Figure 12, where the differential conductance $g = \mathrm{d}I/\mathrm{d}V$ is plotted as a function of gate voltage and bias voltage (cf. Fig.2(b) in the main text), we see the expected loop-shaped structure.

Regime IV can be identified for the gate voltage range $V_{\mathrm{L}} = -2.85$ V to -4 V. In Supplementary Figure 11 we observe the expected high resistance for small bias throughout the full gate voltage range, indicating that, as in the device in the main text, low bias transport is suppressed throughout this regime. For Supplementary Figure 12 we observe diamond shaped regions of suppressed conductance. This means that for each gate voltage transport becomes enabled only above a critical bias voltage. The resulting Diamond shape is a characteristic signature of transport through a charge puddle, as in the device in the main text. The transport gap at small bias voltage, around $\pm 40\ \mu V$, shows that also device 2 exhibits reservoirs with a superconducting gap. The loop-shaped structure visible in regime III is of a similar energy scale as the transport gap in the diamond regime IV. This is also in agreement with the observations described in the main text.

We note that the data presented in Supplementary Figures 11 and 12 were obtained in a current bias configuration, like the data in Fig.2 in the main text. Therefore signatures such as negative differential conductance (NDC) and excited states of the puddle, as they become visible in a voltage bias measurement (cf. Fig. 4 in the main text) do not become visible from these figures. Note that the gate voltage range in which regimes III and IV occur in Supplementary Figures 11 and 12 differ from those in the main text. This is the result of gate history effects as discussed in the main text: Varying the gate voltages many times over a wide range within a single cooling cycle results in a reduced gate action.

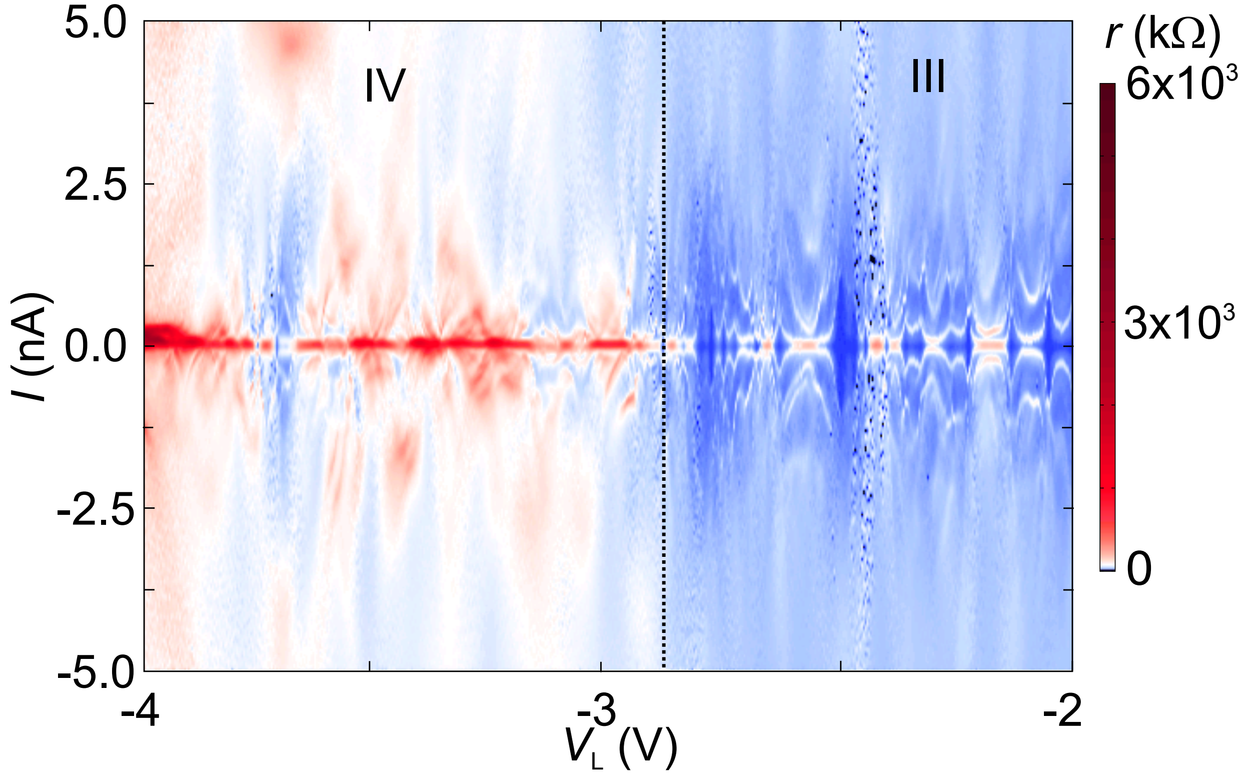

Supplementary Figure 11. **Regime III and IV in Device 2 with differential resistance r as a function of bias current I for different split gate voltages V_{L}** The data are obtained from the device shown in Supplementary Figure 10. Due to gate history effects only gate L is used in this series of measurements. The data are obtained for a different back gate voltage ($V_{\mathrm{BG}} = -9.5V$) compared those shown in Supplementary Figure10 ($V_{\mathrm{BG}} = 0$).

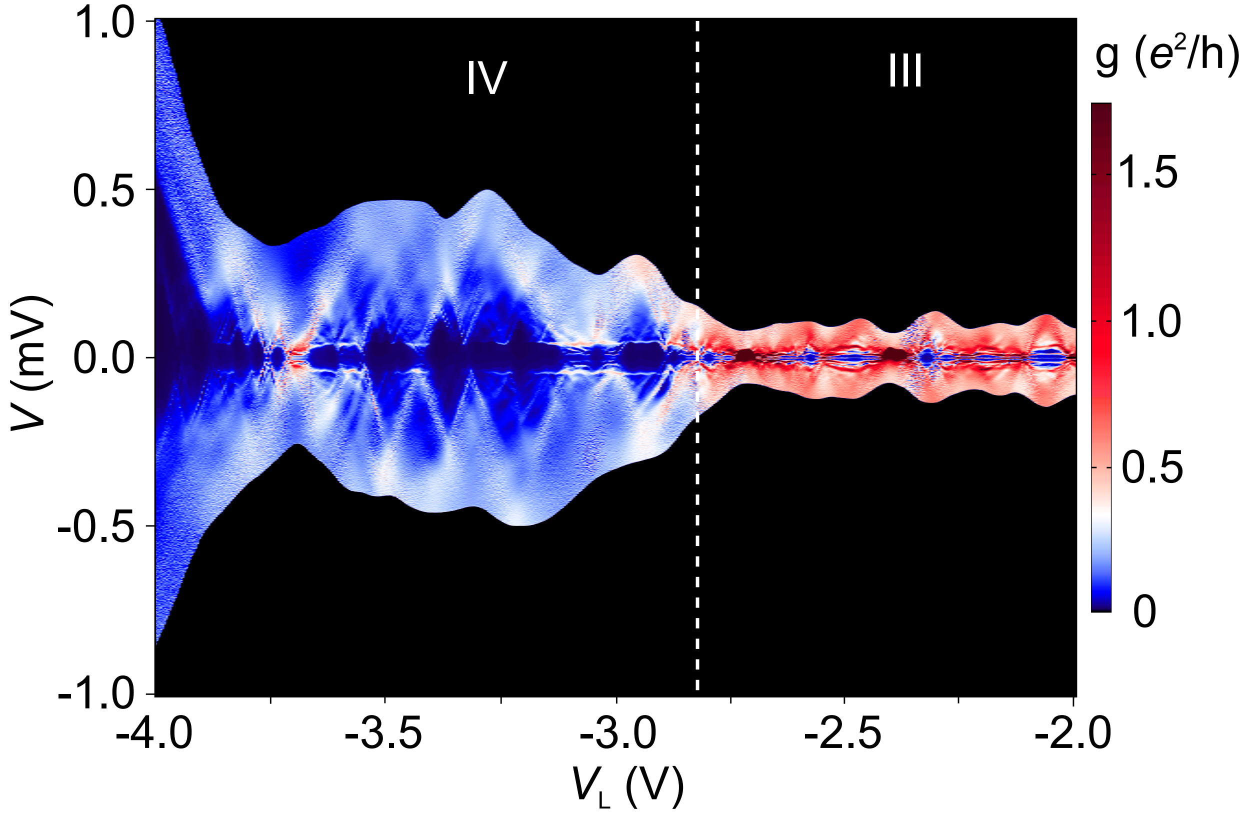

Supplementary Figure 12. **Regime III and IV in Device 2 with differential conductance** g **as a function of bias voltage** V **and split gate voltage** $V_{\mathbf{L}}$ Different presentation of the same data as shown in Supplementary Figure 11.

[1] Blonder, G., Tinkham, M. & Klapwijk, T. Transition from metallic to tunneling regimes in superconducting microconstrictions: Excess current, charge imbalance, and supercurrent conversion. *Phys. Rev. B* **25**, 4515 (1982).

[2] Beenakker, C. & Van Houten, H. Josephson current through a superconducting quantum point contact shorter than the coherence length. *Phys. Rev. Lett.* **66**, 3056 (1991).

[3] Beenakker, C. Three universal mesoscopic josephson effects. In *Transport Phenomena in Mesoscopic Systems*, 235–253 (Springer, 1992).

[4] Monteiro, A. *et al.* Side gate tunable josephson junctions at the $LaAlO_3/SrTiO_3$ interface. *Nano. Lett.* **17**, 715–720 (2017).

[5] Krupka, J., Geyer, R. G., Kuhn, M. & Hinken, J. H. Dielectric properties of single crystals of Al_2O_3, $LaAlO_3$, $NdGaO_3$, $SrTiO_3$, and MgO at cryogenic temperatures. *IEEE Trans. Microwave Theory Techn.* **42**, 1886–1890 (1994).

[6] Landau, L. D., Lífshíts, E. M. & Pitaevskii, L. *Electrodynamics of continuous media*, vol. 8 (Pergamon press Oxford, 1984).

[7] Ang, C. & Yu, Z. dc electric-field dependence of the dielectric constant in polar dielectrics: Multipolarization mechanism model. *Phys. Rev. B* **69**, 174109 (2004).

[8] Stornaiuolo, D. *et al.* Weak localization and spin-orbit interaction in side-gate field effect devices at the $LaAlO_3/SrTiO_3$ interface. *Phys. Rev. B* **90**, 235426 (2014).

[9] Hanson, R., Kouwenhoven, L. P., Petta, J. R., Tarucha, S. & Vandersypen, L. M. Spins in few-electron quantum dots. *Rev. Mod. Phys.* **79**, 1217 (2007).

[10] Zwanenburg, F. A. *et al.* Silicon quantum electronics. *Rev. Mod. Phys.* **85**, 961 (2013).

[11] Hergenrother, J., Tuominen, M. & Tinkham, M. Charge transport by andreev reflection through a mesoscopic superconducting island. *Phys. Rev. Lett.* **72**, 1742 (1994).

[12] Hergenrother, J., Lu, J., Tuominen, M., Ralph, D. & Tinkham, M. Photon-activated switch behavior in the single-electron transistor with a superconducting island. *Phys. Rev. B* **51**, 9407 (1995).

[13] Cheng, G. *et al.* Electron pairing without superconductivity. *Nature* **521**, 196–199 (2015).

[14] Cheng, G. *et al.* Tunable electron-electron interactions in $LaAlO_3/SrTiO_3$ nanostructures. *Phys. Rev. X* **6**, 041042 (2016).

[15] Tomczyk, M. *et al.* Micrometer-scale ballistic transport of electron pairs in $LaAlO_3/SrTiO_3$ nanowires. *Phys. Rev. Lett.* **117**, 096801 (2016).

[16] Kuerten, L. *et al.* In-gap states in superconducting $LaAlO_3/SrTiO_3$ interfaces observed by tunneling spectroscopy. *Phys. Rev. B* **96**, 014513 (2017).
